# Supplementary material for: Study on the Pasting Properties of Indica and Japonica Waxy Rice
Source: Foods. 2022 Apr 14;11(8):1132. doi: 10.3390/foods11081132 (PMC9031608; doi:10.3390/foods11081132)
Supplement: Supplementary file 1 [file foods-11-01132-s001.zip › foods-1650675-supplementary.pdf]

foods

MDPI

Supplementary Materials

Study on the Pasting Properties of Indica and Japonica Waxy Rice

Sicong Fang [1,2,3,4], Cheng Chen [5], Yuan Yao [5], John Nsor-Atindana [6], Fei Liu [1,2,3,4], Maoshen Chen [1,2,3,4,*] and Fang Zhong [1,2,3,4]

[1] State Key Laboratory of Food Science and Technology, Jiangnan University, Wuxi 214122, China; 876072648@qq.om (S.F.); feiliu@jiangnan.edu.cn (F.L.); fzhong@jiangnan.edu.cn (F.Z.)
[2] Science Center for Future Foods, Jiangnan University, Wuxi 214122, China
[3] School of Food Science and Technology, Jiangnan University, Wuxi 214122, China
[4] International Joint Laboratory on Food Safety, Jiangnan University, Wuxi 214122, China
[5] Zhejiang Wufangzhai Industrail Co., Ltd., Jiaxing 310000, China; chencheng@wufangzhai.com (C.C.); yaoyuan@wufangzhai.com (Y.Y.)
[6] Department of Nutrition and Dietetics, School of Allied Health Sciences, University of Health and Allied Sciences, PMB 31, Ho 00233, Ghana; jansor@uhas.edu.gh
* Correspondence: chenmaoshen@jiangnan.edu.cn; Tel.: +86-510-85197579

Table S1. Pasting profiles of ground rice flours.

Sample	Peak viscosity (cP)	Trough viscosity (cP)	Final viscosity (cP)	pasting temperature (℃)
IWR-1-F	1371.83 ± 18.08^{i}	859.68 ± 29.34^{g}	1125.39 ± 7.08^{g}	72.39 ± 0.08^{h}
IWR-2-F	1242.29 ± 33.31^{e}	833.64 ± 31.61^{e}	1134.20 ± 37.76^{h}	72.39 ± 0.02^{h}
IWR-3-F	1329.82 ± 21.37^{h}	899.50 ± 10.82^{i}	1124.11 ± 19.43^{f}	70.33 ± 0.02^{f}
IWR-4-F	1297.98 ± 28.99^{g}	855.49 ± 24.03^{f}	1116.36 ± 29.37^{e}	70.31 ± 0.01^{e}
IWR-5-F	1277.24 ± 14.52^{f}	877.31 ± 5.59^{h}	1174.09 ± 50.35^{i}	70.34 ± 0.03^{g}
JWR-1-F	904.32 ± 20.06^{d}	652.04 ± 2.69^{c}	854.66 ± 14.01^{c}	68.32 ± 0.04^{d}
JWR-2-F	900.48 ± 3.40^{c}	669.05 ± 1.99^{d}	855.58 ± 2.57^{d}	66.27 ± 0.04^{c}
JWR-3-F	667.03 ± 16.94^{a}	496.62 ± 15.31^{a}	656.60 ± 6.60^{a}	64.21 ± 0.07^{a}
JWR-4-F	764.90 ± 13.33^{b}	502.73 ± 3.69^{b}	727.15 ± 0.39^{b}	64.26 ± 0.00^{b}

"IWR" stands for indica waxy rice; "JWR" stands for japonica waxy rice; Values are showed by Mean ± SD and values, different letters within a column indicate significant differences between mean values ($p < 0.05$).

Table S2. Pasting profiles of rice flours after removing lipid and protein.

Sample	Peak viscosity(cP)	Trough viscosity (cP)	Final viscosity (cP)	pasting temperature (℃)
IWR-1-L	1529.04 ± 11.28^{i}	906.11 ± 10.58^{i}	1262.91 ± 5.15^{i}	70.34 ± 0.03^{d}
IWR-2-L	1435.56 ± 5.12^{g}	832.94 ± 16.56^{f}	1141.39 ± 34.03^{f}	70.37 ± 0.00^{e}
IWR-3-L	1494.72 ± 22.96^{h}	784.92 ± 51.91^{e}	1116.70 ± 20.99^{e}	70.34 ± 0.07^{d}
IWR-4-L	1428.86 ± 24.58^{e}	866.76 ± 40.16^{g}	1212.15 ± 36.03^{h}	70.39 ± 0.00^{f}
IWR-5-L	1444.92 ± 4.53^{g}	885.61 ± 25.67^{h}	1188.35 ± 32.62^{g}	70.36 ± 0.07^{e}
JWR-1-L	1099.72 ± 21.09^{d}	638.80 ± 15.86^{c}	900.34 ± 7.48^{b}	68.31 ± 0.04^{c}
JWR-2-L	984.44 ± 22.78^{b}	655.27 ± 23.91^{d}	943.25 ± 20.55^{d}	66.24 ± 0.06^{b}
JWR-3-L	916.76 ± 31.15^{a}	559.88 ± 25.83^{a}	810.34 ± 20.29^{a}	64.21 ± 0.00^{a}
JWR-4-L	1045.02 ± 3.11^{c}	629.00 ± 20.27^{b}	910.19 ± 10.51^{c}	64.23 ± 0.02^{a}
IWR-1-L-P	1632.76 ± 113.05^{i}	1003.64 ± 77.58^{h}	1181.24 ± 45.45^{e}	72.44 ± 0.02^{i}
IWR-2-L-P	1574.11 ± 101.34^{f}	1060.01 ± 17.20^{i}	1202.78 ± 25.08^{h}	72.43 ± 0.01^{h}
IWR-3-L-P	1543.42 ± 36.22^{e}	942.94 ± 38.79^{f}	1113.94 ± 21.14^{b}	70.33 ± 0.05^{e}
IWR-4-L-P	1585.89 ± 112.12^{g}	972.80 ± 29.48^{g}	1271.42 ± 5.52^{i}	72.38 ± 0.01^{g}
IWR-5-L-P	1543.28 ± 4.06^{d}	925.92 ± 54.54^{e}	1189.41 ± 43.81^{g}	70.40 ± 0.01^{f}
JWR-1-L-P	1375.78 ± 10.40^{b}	861.92 ± 47.24^{d}	1185.00 ± 44.21^{f}	68.27 ± 0.03^{c}

JWR-2-L-P	1391.56 ± 52.54c	805.76 ± 32.35b	1141.20 ± 12.45c	68.30 ± 0.00d
JWR-3-L-P	1464.09 ± 3.18d	751.33 ± 12.09a	1047.25 ± 12.24a	66.18 ± 0.02a
JWR-4-L-P	1591.83 ± 7.32h	847.02 ± 13.60c	1150.19 ± 27.32d	66.26 ± 0.00b

"IWR" stands for indica waxy rice; "JWR" stands for japonica waxy rice. Values are showed by Mean ± SD and values; Different letters within a column of each index indicate significant differences between mean values (n=3) ($p < 0.05$).

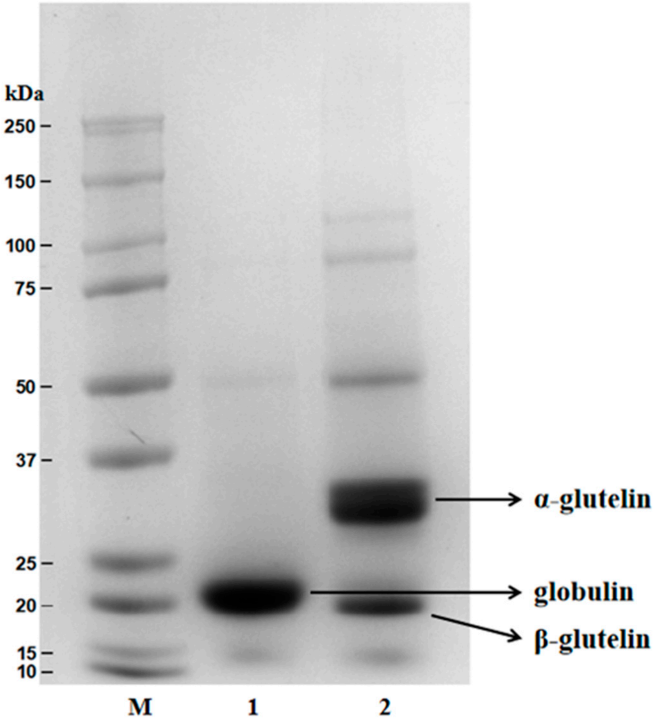

Figure S1. The SDS-PAGE of protein fractions. M: Marker; 1: extracted by 5% NaCl solution; 2: extracted by 0.02M NaOH solution.

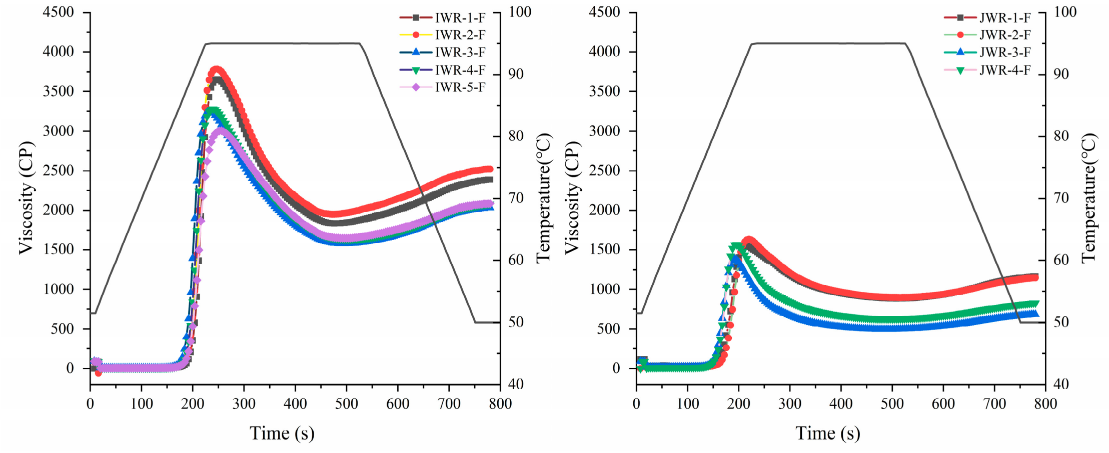

Figure S2. The pasting characteristics determined by RVA of five IWR flours (a) and four JWR flours (b). "IWR" stands for indica waxy rice; "JWR" stands for japonica waxy rice. "F" stands for flour.

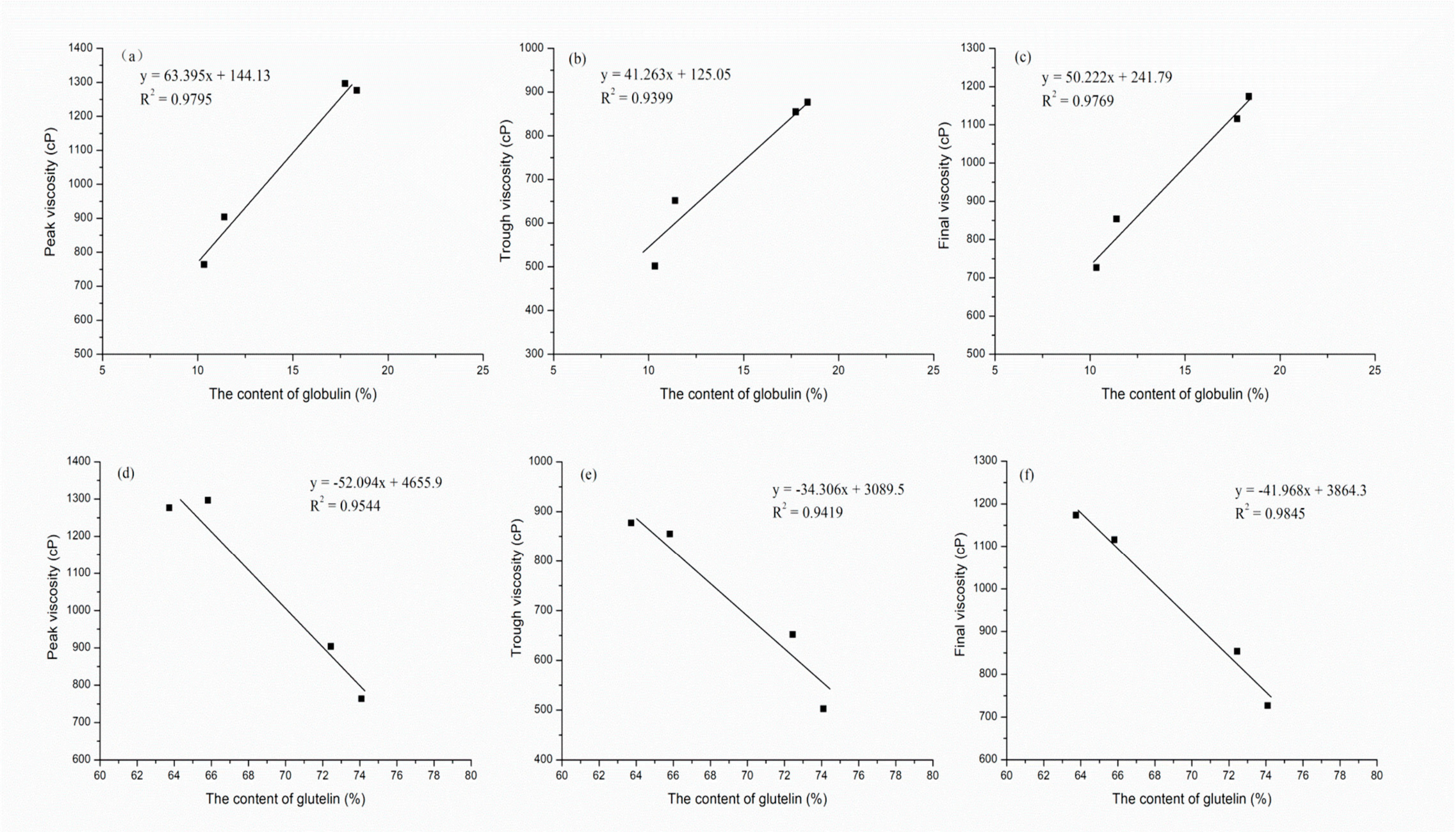

Figure S3. The relationship between peak viscosity (a, d), trough viscosity (b, e) and final viscosity (c, f) and the content of globulin (a-c) and glutelin (d-f) of waxy rice flour.
